# Current Pathologic Scoring Systems for Metal-on-metal THA Revisions are not Reproducible

**DOI:** 10.1007/s11999-017-5432-4

**Published:** 2017-07-07

**Authors:** Christiaan Smeekes, Arjen H. G. Cleven, Bart C. H. van der Wal, Stefan V. Dubois, Remigio W. Rouse, Bastiaan F. Ongkiehong, Ron Wolterbeek, Rob G. H. H. Nelissen

**Affiliations:** 1 Department of Orthopaedics, Leiden University Medical Center, PO Box 9600, 2300 RC Leiden, The Netherlands; 2 Department of Pathology, Leiden University Medical Centre, Leiden, The Netherlands; 3 Department of Orthopaedics, Meander Medical Centre, Amersfoort, The Netherlands; 4 Department of Pathology, Meander Medical Centre, Amersfoort, The Netherlands; 5 Department of Medical Statistics and Bioinformatics, Leiden University Medical Centre, Leiden, The Netherlands

## Abstract

**Background:**

The aseptic lymphocyte vasculitis-associated lesion (ALVAL) score and the modified Oxford ALVAL score are frequently used scoring methods to evaluate the morphologic features of periprosthetic tissues around metal-on-metal (MoM) hip implants. Except for the initial studies of these two morphology scoring methods, to our knowledge, no other studies have reported on intraclass correlation coefficient (ICC) values for interobserver reliability of these scoring methods.

**Questions/purposes:**

Are the ALVAL and Oxford ALVAL scores reproducible?

**Methods:**

The periprosthetic tissue of 37 revisions of 36 patients with failed MoM THAs were independently scored by three experienced pathologists using ALVAL and Oxford ALVAL scoring methods. All patients were included who underwent revision surgery in our hospital until January 2013, with a large-head MoM prosthesis and also met the criteria: blood serum cobalt levels, available MRI scan, and intraarticular cobalt levels. The population included 26 patients with pseudotumors diagnosed by two radiologists using the method described by Matthies et al. The ALVAL describes morphologic features of the synovial lining, tissue organization, and inflammatory cell infiltrate in periprosthetic tissues. The Oxford-ALVAL score uses a semiquantitative measure of the immune response which should be easier to score.

**Results:**

The ALVAL score showed an ICC of 0.38 (95% CI, 0.18–0.58) (fair) for the sum score and this improved up to 0.50 (95% CI, 0.31–0.68) (moderate) using the modified Oxford ALVAL score. The individual parameters of the ALVAL score showed an ICC for the scoring of inflammatory infiltrate of 0.37 (95% CI, 0.17–0.57), an ICC of 0.32 (95% CI, 0.12–0.53) for the scoring of tissue organization, and an ICC of 0.14 (95% CI, −0.04 to 0.34) for synovial lining.

**Conclusions:**

Scoring morphologic features of MoM tissue is not reproducible using the ALVAL score or the Oxford ALVAL score. This may reflect heterogeneous morphologic features in tumor tissue and between different tumor tissue samples that cannot be reliably quantified by pathologists using the parameters of these two scoring methods. An alternative, simplified scoring system should be developed to improve the interrater agreement.

**Level of Evidence:**

Level III, diagnostic study.

**Electronic supplementary material:**

The online version of this article (doi:10.1007/s11999-017-5432-4) contains supplementary material, which is available to authorized users.

## Introduction

Despite hopes that metal-on-metal (MoM) bearings would provide long-lasting pain relief and restoration of function in THAs, revision rates for many designs have been alarmingly high. Release of metal ions and particles from the MoM bearing leads to elevated high local and systemic exposure to cobalt and chromium ion levels. At the local level, pseudotumor is a frequent finding, described as development of a cystic solid mass in the periarticular region, which has a direct communication with the joint [14]. A possible explanation for the occurrence of pseudotumors and failure of the MoM THA is the toxicity of the local metal debris rich in cobalt particles that can induce DNA damage and cell death, which occurs either by disruption of the membrane or because of the DNA damage. An inflammatory mass develops in response to the cytokines released [10]. Although pseudotumors also are seen in patients after conventional THA with ceramic-on-polyethylene [3] and are described in case reports of metal-on-polyethylene [17, 21], risk for development of these pseudotumors is increased in patients with elevated serum metal ion levels [4].

Aseptic lymphocyte vasculitis-associated lesion (ALVAL), first reported by Davies et al. [8], is a histologic description made from tissue sampling at the time of surgery identifying an abundance of lymphocytes in the local pericapsular tissue. ALVAL typically is associated with local metal ion release. A meta-analysis showed a pooled estimate of the incidence of pseudotumor or ALVAL in MoM hip articulations to be 0.6% [30], and another study showed up to 6.5% ALVAL [16]. The most-used description method of periprosthetic tissues around MoM hip implants is the ALVAL score of Campbell et al. [7]. This subsequently was modified by Grammatopoulos et al. [12], (herein referred to as the Oxford ALVAL) to be able to distinguish if the inflammatory changes and tissue necrosis seen in periprosthetic tissues around failed MoM hip resurfacing implants are attributable to cytotoxicity or hypersensitivity tissue necrosis, and the extent of the inflammatory cell infiltrate was included. Both scoring systems are widely used [6, 9, 15, 22–24, 26, 27], however to our knowledge, other than the initial studies [7, 12], no other studies have reported on interrater reliability. Thus, it is unclear if these scoring instruments are reproducible.

We therefore asked whether the ALVAL and Oxford ALVAL scores were reproducible.

## Patients and Methods

Between February 2008 and January 2011, a series of 377 uncemented primary MoM THAs with a M2a-38™ and Taperloc® stem combination (Biomet, Warsaw, IN, USA) were performed at the Meander Medical Centre. During that period, we used this implant when there was an indication for a THA. Of the patients who were treated with this approach, nine patients (3%) had died, three (1%) were lost to followup, and four (1%) underwent revision surgery before the screening protocol (two infections, one periprosthetic fracture, and one because of pain and subluxations). Three hundred thirty-five patients (361 hips; 95%) were available for followup at a minimum of 11 months (mean, 30 months; range, 11–58 months) [28]. After the first concerns of MoM THA and an alert issued by the Dutch Orthopaedic Association, all patients were subjected to a screening protocol. For the current study, patients who underwent revision surgery because of failure of their MoM hip prostheses were included. A total of 71 revisions were performed in 70 patients. Twenty revisions were not MoM related. Fifty-one revisions were related to MoM problems. Of these, 36 patients with 37 revisions (one bilateral) were selected for the current study because tissue samples, intraarticular cobalt values, and MR images were available. One patient had bilateral MoM THA and underwent revision on both sides; 10 patients had bilateral MoM THAs and underwent revision on one side; and all other patients underwent revision on their unilateral MoM THA. The mean age of the patients at primary surgery was 62 years (SD, 8.2 years); 29 patients were women. The main reason for primary surgery was osteoarthritis (Table 1). The mean serum cobalt level was 20 µg/L (SD, 33 µg/L) and the mean intraarticular fluid cobalt was 2240 µg/L (SD, 2689 µg/L) (Table 1). Pain was reported by 28 patients (76%).

**Table 1:** Clinical data

Demographics	Mean (SD)
Mean age at primary surgery (years)	62 (8)
Sex	
Male	8 (22%)
Female	29 (78%)
BMI (kg/m^2^)	28 (4)
Time until revision surgery (months)	36 (9)
Reasons for surgery	
Osteoarthritis	33 (89%)
Secondary osteoarthritis	3 (8%)
Necrosis of the femoral head	1 (3%)
Serum cobalt (µg/L)	20 (33)
Intraarticular cobalt (µg/L)	2240 (2689)
Pseudotumor classification [18]	
0	0
1	0
2A	24
2B	0
3	2

Twenty-six pseudotumors were diagnosed on MRI. Most of the pseudotumors were described as 2A according to the classification described by Matthies et al. [18] (n = 24). Two Type 3 pseudotumors were diagnosed (Table 1). Reasons for revision were pseudotumor formation in combination with pain and elevated serum levels of cobalt or pain and elevated serum cobalt levels without pseudotumor formation and failure of the hip for other reasons (acetabular loosening [n = 2] and component impingent [n = 1]; these patients also had elevated cobalt levels). During revision surgery two to three samples were taken by the surgeon of the spots which were macroscopically affected by MoM disease. Each sample was formalin-fixed, paraffin-embedded, and sectioned. Slides were stained with standard hematoxylin and eosin. Sample slides (three to four for each patient) were independently examined by three pathologists (AHGC, RWR, SVD) who were experienced in diagnosing skeletal and soft tissue related diseases, and thus well trained in recognizing different types of inflammation cells and patterns of inflammation. These pathologists independently evaluated the tissue samples using the ALVAL score [7] and the adapted Oxford ALVAL scoring method [12]. The total scores of each pathologist are shown in a supplemental appendix (Appendix 1. Supplemental materials are available with the online version of *CORR* ^®^.) that shows the distribution of low, moderate, or high ALVAL scores were comparable among the pathologists. The slides were scored with the ALVAL score as described by Campbell et al. [7] and the modifications of the Oxford ALVAL by Grammatopoulos et al. [12] (Table 2). All three pathologists were blinded to the clinical outcome. The intraclass correlation coefficient (ICC) was obtained from the individual parameter scores.

**Table 2:** Scoring of the histologic findings

Scoring	Points
Synovial lining (ALVAL)	
Intact synovial lining	0
Focal loss of synovial surface, fibrin attachment may occur	1
Moderate to marked loss of synovial surface, fibrin attachment	2
Complete loss of synovium, abundant attached fibrin and/or necrosis of lining tissue	3
Inflammatory infiltrate (ALVAL)	
Minimal inflammatory cell infiltrates	0
Predominantly macrophages, occasional lymphocytes may occur	1
Mix of macrophages and lymphocytes, either diffuse and/or small (< 50% of hpf) perivascular aggregates	2
Mix of macrophages and lymphocytes, large (> 50% hpf) perivascular aggregates may occur	3
Predominantly lymphocytes, mostly in multiple, large (> 50% hpf) perivascular aggregates, follicles may be present	4
Tissue organization (ALVAL)	
Normal tissue arrangement	0
Mostly normal tissue arrangement, small areas of synovial hyperplasia, focal necrosis may occur	1
Marked loss of normal arrangement, appearance of distinct cellular and acellular zones, thick fibrous layers may occur	2
Perivascular lymphocytic aggregates mostly located distally, thick acellular areas may occur	3
Inflammatory cells (macrophages), (lymphocytes), (plasma cells), (eosinophil polymorphs) (Oxford ALVAL)	
Absent	0
Few	1+
Many	2+
Abundant	3+
Necrosis (Oxford ALVAL)	
Absent	0
Scattered small necrotic areas	1+
Frequent small or large necrotic areas with up to 25% tissue involvement	2+
Extensive necrosis with > 25% tissue necrosis	3+
Oxford ALVAL score (semiquantitative score)	
No evidence of a perivascular lymphocyte infiltrate	0
Little evidence of a perivascular lymphocytic infiltrate with lymphocyte cuffing of blood vessels being fewer than five cells in thickness	1
Several perivascular lymphoid aggregates with lymphocyte cuffing of vessels being five to 10 cells in thickness	2
Numerous large perivascular lymphoid aggregates with lymphocyte cuffing around vessels being more than 10 cells in thickness	3

The original ALVAL score [7] uses the first three categories (synovial lining, inflammatory infiltrate, and tissue organization); the Oxford scoring system [12] assesses tissue necrosis and the extent of the inflammatory cell infiltrate in the periprosthetic tissues. The presence of specific inflammatory cells (macrophages, lymphocytes, plasma cells, eosinophil polymorphs) was noted, and the presence or absence of an ALVAL response was assessed semiquantitatively as previously described. In the current study all parameters are scored; the number of specific inflammatory cells is scored as 0 (absent), 1 (few), 2 (many), or 3 (abundant). Necrosis was scored as 0 (absent), 1 (scattered small necrotic areas), 2 (frequent small or large necrotic areas with up to 25% tissue involvement), or 3 (extensive necrosis with > 25% tissue involvement); ALVAL = aseptic lymphocyte vasculitis-associated lesion; hpf = high-power field.

The scientific committee of the Leiden University Medical Centre and the ethical committee in the Meander Medical Centre waived approval for the human protocol for this investigation, because the removed tissue was sent for routine histopathologic analysis. Because revision surgery had to be performed at such a short followup and because scientific concerns were present regarding the tissue reactions potentially caused by the MoM articulation, performing a histopathologic analysis was considered part of good clinical practice.

During the outpatient clinic visit, patients answered a standard clinical questionnaire (pain: yes or no) and underwent a physical examination. Blood samples were collected in a metal-free container. Serum cobalt was determined with the use of an Aanalyst^TM^ 800 Atomic Absorption Spectrophotometer (PerkinElmer, Waltham, MA, USA). Cobalt serum levels between 0.04 and 0.64 µg/L were considered normal in the general population [11]. In case of revision surgery, a sample of the intraarticular fluid was taken and the cobalt values of the fluid were determined using the AAnalyst^TM^ 800 Atomic Absorption Spectrophotometer.

A contrast-enhanced MRI of the hip region with metal artifact reducing sequences (MARS) was performed on patients with osteolysis observed on the radiograph, elevated cobalt levels greater than 5 µg/L (cutoff value in patients with a MoM implant [13]), or with pain. Pain was defined as either the presence or absence of any pain in the hip area reported by the patient. Patients who met these criteria received routine annual followup. A 1.5-T MRI unit (Achieva; Philips Healthcare, Best, The Netherlands) was used to obtain the MARS sequences. As a contrast agent, Dotarem^®^ (Guerbet, Paris, France) was used.

All MRI scans were evaluated by a senior musculoskeletal radiologist (MN) and a resident in radiology (BS) with expertise in musculoskeletal disease. The criteria of the Anderson et al. [2], Hauptfleisch et al. [14], and Matthies et al. [18] classifications were used. These criteria were periprosthetic soft tissue mass or fluid-filled periprosthetic cavities and their diameter; the thickness and regularity of the wall; muscle atrophy; edema or bone marrow edema, and tendon avulsion or fracture of the bone. The classification of Anderson et al. [2] is based on their experience regarding how the MRI appeared to influence management of patients with a pseudotumor. The classifications of Matthies et al. [18] and Hauptfleisch et al. [14] are based on radiologic findings to classify the pseudotumor. In the results, the classification of Matthies et al [18] was used to describe the findings because it provided the best ICC (0.49) in our cohort.

The original ALVAL scoring system described by Campbell et al. [7] uses three different histologic criteria: synovial lining, inflammatory infiltrate, and tissue organization, which add up to an overall score. The modified Oxford ALVAL scoring system described by Grammatopoulos et al. [12] adds tissue necrosis and the extent of the inflammatory cell infiltrate in the periprosthetic tissues. The presence of specific inflammatory cells (macrophages, lymphocytes, plasma cells, eosinophil polymorphs) is noted and the ALVAL response is rated semiquantitatively (Table 2).

### Statistical Analysis

Descriptive analyses were performed on final outcomes. The results are expressed as means with SD or medians with ranges where relevant.

The interobserver reliability was calculated as an ICC with a 95% CI based on a two-way random-ANOVA with patient and pathologist as random factors for three pathologists. This ICC has an interpretation as a weighted kappa with quadratic weights.

The ICC value for agreement was interpreted as follows: poor < 0.20; fair, 0.21 to 0.40; moderate, 0.41 to 0.60; good, 0.61 to 0.80; and very good, 0.81 to 1.0 [5]. SPSS Statistics Version 20.0 (IBM Corporation, Armonk, NY, USA) was used for the analysis.

## Results

**Fig. 1A–B:**
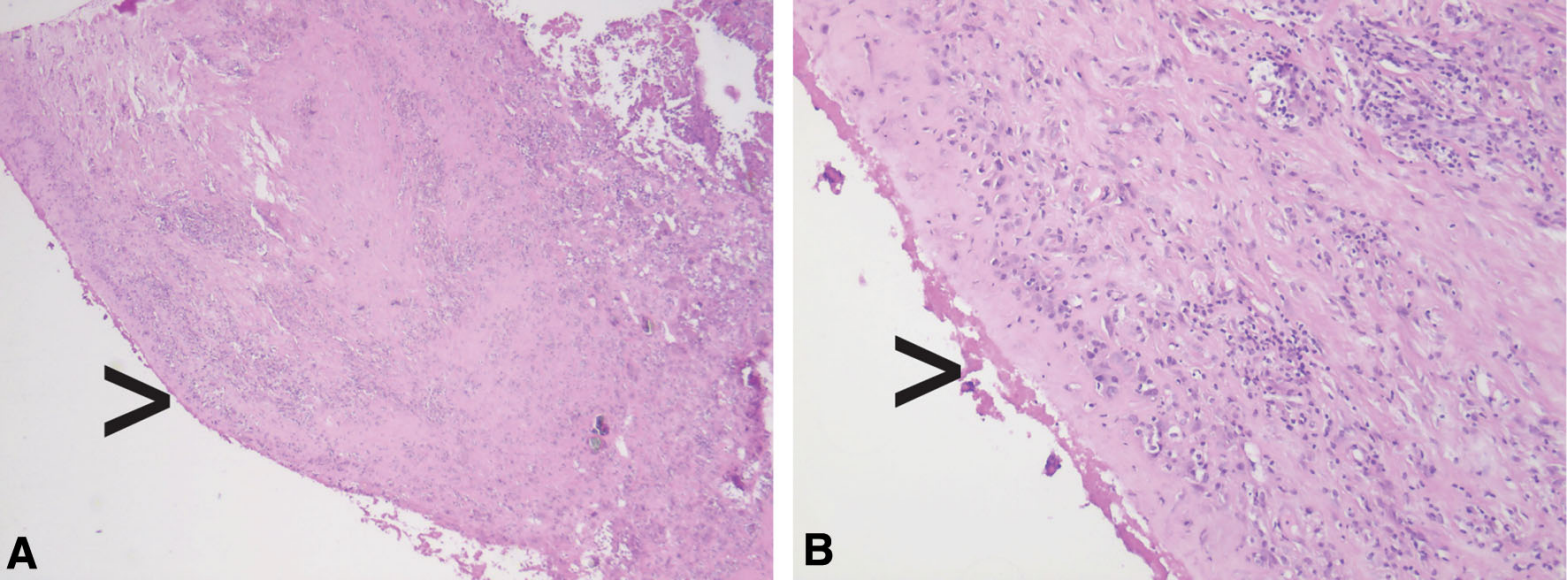
Histologic analyses of hematoxylin and eosin-stained sections at **(A)** ×2.5 magnification and **(B)** ×10 magnification show the morphologic spectrum in discordant cases with no dense lymphocytic infiltrate and areas with no intact synovial lining with fibrin attachment (black arrows).

**Fig. 2A–B:**
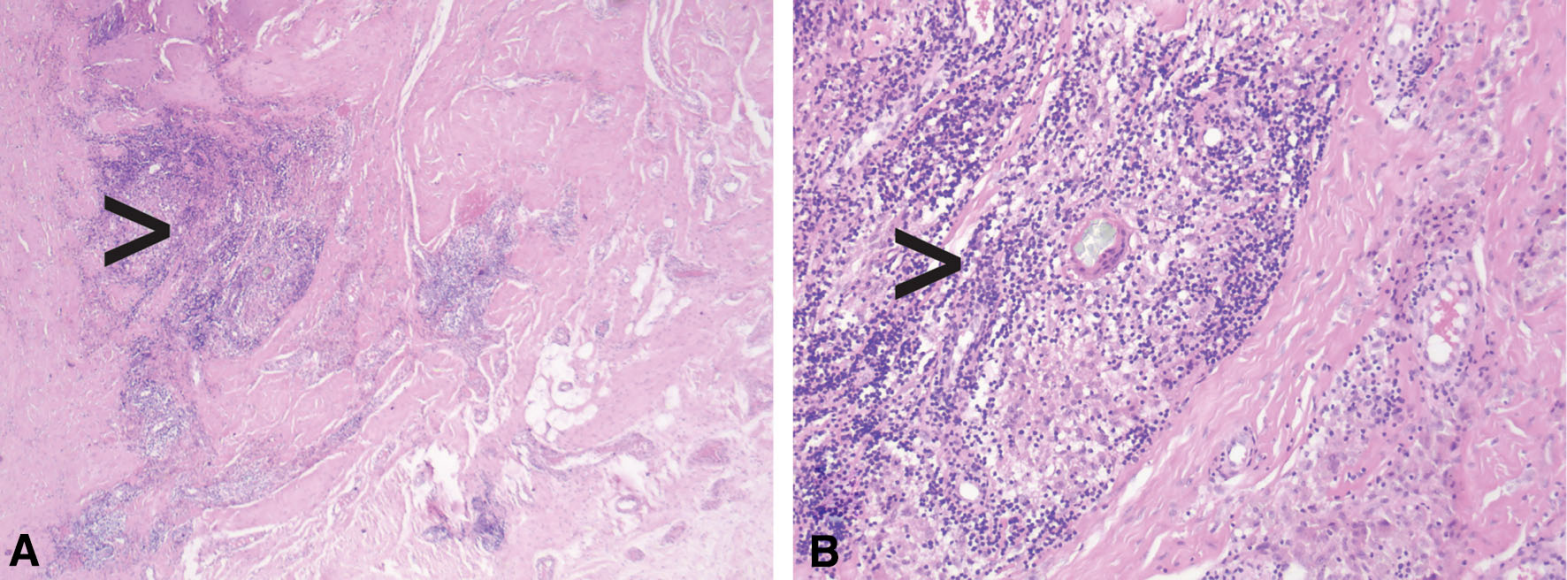
Histologic analyses of hematoxylin and eosin-stained sections at **(A)** ×2.5 magnification and **(B)** ×10 magnification show discordant cases with dense perivascular lymphocytic aggregates (black arrows).

The ICC for the sum score using the ALVAL classification is 0.38 (95% CI, 0.18–0.58), which is categorized as fair. The individual parameters of this score show an ICC for the scoring of inflammatory infiltrate of 0.37 (95% CI, 0.17–0.57), an ICC of 0.32 (95% CI, 0.12–0.53) for the scoring of tissue organization, and an ICC of 0.12 (95% CI, 0.00–0.34) for synovial lining (Table 3). The ICC for the sum score using the Oxford ALVAL score is 0.50 (95% CI, 0.30–0.68), which is categorized as moderate. The scoring of inflammatory cells and necrosis showed ICC between 0.04 (95% CI, 0.00–0.24) and 0.50 (95% CI, 0.29–0.68). The highest ICC, 0.50 (95% CI, 0.29–0.68) was found for inflammatory cells (lymphocytes) (Table 3). Heterogeneous morphologic features in a discordant case with no dense lymphocytic infiltrate and areas with no intact synovial lining with fibrin attachment (Fig. 1) and in a discordant case with dense perivascular lymphocytic aggregates (Fig. 2) are shown.

**Table 3:** Intraclass correlation coefficients of the morphologic features of the scoring

Morphologic features	Intraclass correlation (95% CI)
Synovial lining	0.12 (0.00–0.34)
Inflammatory infiltrate	0.37 (0.17–0.57)
Tissue organization	0.32 (0.12–0.53)
Sum score	0.38 (0.18–0.58)
Inflammatory cells (macrophages)	0.44 (0.24–0.64)
Inflammatory cells (lymphocytes)	0.50 (0.29–0.68)
Inflammatory cells (plasma cells)	0.29 (0.09–0.50)
Inflammatory cells (eosinophil polymorphs)	0.04 (0.00–0.24)
Necrosis	0.37 (0.17–0.58)
Oxford ALVAL score	0.50 (0.30–0.68)

ALVAL = aseptic lymphocyte vasculitis-associated lesion.

## Discussion

MoM THAs have a high failure rate [29]. Elevated serum cobalt levels, pseudotumors, and tissue reaction have been described [13, 14, 31]. Pathologic findings in patients with failed MoM THAs have been described using the ALVAL and Oxford ALVAL scoring methods [7, 12]. Only the initial studies [7, 12] report ICC values for interobserver reliability. In the current study, we tested the reproducibility of these scoring systems by three independent pathologists. The scoring system of Campbell et al. [7] showed an ICC of 0.38 (95% CI, 0.18–0.58) for the sum score, which is rated as fair. The sum score improved up to 0.5 (95% CI, 0.30–0.68) using the modified Oxford ALVAL score [12].

This study had several limitations. Only one type of implant was used, which might not be characteristic of other MoM devices. The selection for revision surgery was made by using the described screening method. All patients who underwent revision surgery were symptomatic and most of the patients had high cobalt serum levels. Thus, our findings may not be applicable to patients with different presentations, such as asymptomatic patients with concerning MRI and laboratory findings. No prelearning meeting with all three pathologists was done to describe how to score the tissue slides using the scoring methods. Nevertheless all pathologists are experienced in diagnosing skeletal and soft tissue-related diseases, and thus well trained in recognizing different types of inflammation cells and patterns of inflammation. We believe that the poor ICCs we found in our study regarding the ALVAL and Oxford ALVAL scores are attributable to the complex, and therefore not reproducible, scoring methods rather than expert level of individual pathologists. We had a relatively small sample size, meaning that we might not have detected a truly high level of reliability. However, the studies reporting the original ALVAL [7] and Oxford ALVAL [12] scores were based on 32 and 65 samples, respectively.

Although the modified classification system improves the ICC value, it is still no more than moderate. A moderate score indicates inadequate interrater agreement and study results are not reliable to draw any definitive conclusions [5, 19]. Our low ICC values for the individual parameters (inflammatory cells and necrosis) varying between 0.04 and 0.50 underline the low reproducibility of these morphologic findings. In contrast to our results, Campbell et al. [7] reported an interrater reliability of 0.71 and Grammatopoulos et al. [12] reported interrater reliability of 0.74. The ICCs of the ALVAL and the Oxford ALVAL was scored by two observers in these original studies.

Despite that the ALVAL and Oxford ALVAL scoring methods are not well validated, these scoring systems were used in other studies without reporting ICC values [6, 9, 15, 22^_^24, 26, 27]. These study results should be interpreted with caution. Our results clearly illustrate that the ALVAL and Oxford ALVAL scoring systems are not reproducible in our hands, and therefore we believe that clinicians should not use these scoring methods. Larger cohorts are required for the development of an alternative, more-simplified scoring method. Multiple pathologists should score a set of cases to investigate how well the new scoring method is reproducible. Digital imaging analysis showed good results in liver fibrosis [25], in assessing digital ulcers in patients with systemic sclerosis [1], and in analysis of cancer stem cell marker expression [20]. This type of tissue analysis might be a good alternative for scoring of MoM periprosthetic tissue.

If this scoring method is reproducible, correlation with clinically meaningful data should be performed.

## Electronic supplementary material

Below is the link to the electronic supplementary material.

Supplementary material 1 (DOCX 26 kb)
