# Supplementary material for: Current Pathologic Scoring Systems for Metal-on-metal THA Revisions are not Reproducible
Source: Clin Orthop Relat Res. 2017 Jul 7;475(12):3005–11. doi: 10.1007/s11999-017-5432-4 (PMC5670057; doi:10.1007/s11999-017-5432-4)
Supplement: Supplementary file 1 — Supplementary material 1 (DOCX 26 kb) [file 11999_2017_5432_MOESM1_ESM.docx]

**Appendix 1**. Scores of each pathologist

|  | Synovial lining | Inflammatory infiltrate | Tissue organization | ALVAL | Macrophages | Lymphocytes | Plasma cells | Eosinophil polymorphs | Necrosis | Oxford ALVAL (semiquantitative score) |
| --- | --- | --- | --- | --- | --- | --- | --- | --- | --- | --- |
|  | 0-3 | 0-4 | 0-3 | Sum score | 0-3 | 0-3 | 0-3 | 0-3 | 0-3 | 0-3 |
| Patient number  Pathologist 1 |  |  |  |  |  |  |  |  |  |  |
| 1 | 3 | 3 | 3 | 9 | 3 | 2 | 1 | 1 | 3 | 1 |
| 2 | 3 | 3 | 3 | 9 | 3 | 2 | 1 | 0 | 2 | 3 |
| 3 | 2 | 2 | 1 | 5 | 2 | 2 | 1 | 0 | 2 | 1 |
| 4 | 3 | 3 | 3 | 9 | 2 | 2 | 1 | 0 | 2 | 2 |
| 5 | 3 | 1 | 2 | 6 | 2 | 1 | 0 | 0 | 1 | 1 |
| 6 | 3 | 2 | 3 | 8 | 2 | 2 | 1 | 0 | 3 | 2 |
| 7 | 3 | 2 | 3 | 8 | 2 | 2 | 0 | 0 | 1 | 2 |
| 8 | 3 | 1 | 1 | 5 | 3 | 2 | 0 | 0 | 1 | 1 |
| 9 | 3 | 4 | 2 | 9 | 3 | 3 | 1 | 1 | 1 | 3 |
| 10 | 3 | 1 | 2 | 6 | 3 | 1 | 0 | 0 | 2 | 1 |
| 11 | 3 | 2 | 2 | 7 | 3 | 2 | 2 | 0 | 2 | 2 |
| 12 | 3 | 2 | 2 | 7 | 2 | 2 | 1 | 0 | 3 | 1 |
| 13 | 3 | 1 | 2 | 6 | 3 | 1 | 0 | 0 | 2 | 1 |
| 14 | 3 | 1 | 2 | 6 |  | 2 | 0 | 0 | 3 | 1 |
| 15 | x | x | x | x | x | x | x | x | x | x |
| 16 | 3 | 2 | 2 | 7 | 1 | 2 | 0 | 0 | 2 | 2 |
| 17 | 2 | 4 | 3 | 9 |  | 3 | 1 | 1 | 1 | 3 |
| 18 | 3 | 4 | 3 | 10 | 2 | 3 | 0 | 1 | 1 | 3 |
| 19 | 3 | 3 | 3 | 9 | 3 | 2 | 0 | 0 | 2 | 3 |
| 20 | 3 | 1 | 3 | 7 | 0 | 1 | 0 | 0 | 2 | 1 |
| 21 | 3 | 2 | 3 | 8 | 3 | 2 | 1 | 0 | 1 | 2 |
| 22 | 3 | 1 | 2 | 6 | 2 | 1 | 0 | 1 | 2 | 1 |
| 23 | 3 | 2 | 2 | 7 | 2 | 2 | 1 | 0 | 1 | 2 |
| 24 | 3 | 2 | 2 | 7 | 3 | 2 | 0 | 0 | 3 | 2 |
| 25 | 3 | 2 | 2 | 7 | 2 | 1 | 0 | 0 | 1 | 2 |
| 26 | 3 | 1 | 2 | 6 | 3 | 1 | 0 | 0 | 1 | 1 |
| 27 | 3 | 1 | 2 | 6 | 2 | 1 | 0 | 0 | 2 | 1 |
| 28 | 3 | 2 | 3 | 8 | 2 | 2 | 0 | 0 | 2 | 2 |
| 29 | 3 | 1 | 3 | 7 | 2 | 2 | 1 | 1 | 2 | 1 |
| 30 | 3 | 2 | 2 | 7 | 2 | 2 | 1 | 0 | 2 | 1 |
| 31 | 3 | 2 | 2 | 7 | 3 | 2 | 1 | 1 | 3 | 1 |
| 32 | 3 | 3 | 2 | 8 | 3 | 3 | 1 | 0 | 1 | 2 |
| 33 | 3 | 1 | 1 | 5 | 3 | 1 | 1 | 1 | 1 | 1 |
| 34 | 3 | 3 | 3 | 9 | 3 | 2 | 1 | 0 | 2 | 2 |
| 35 | 3 | 1 | 1 | 5 | 3 | 1 | 0 | 0 | 2 | 1 |
| 36 | 3 | 2 | 2 | 7 | 3 | 2 | 0 | 0 | 1 | 1 |
| 37 | 3 | 3 | 2 | 8 | 3 | 3 | 1 | 0 | 2 | 3 |

|  |  |  |  |  |  |  |  |  |  |  |
| --- | --- | --- | --- | --- | --- | --- | --- | --- | --- | --- |
| Pathologist 2 |  |  |  |  |  |  |  |  |  |  |
|  |  |  |  |  |  |  |  |  |  |  |
| 1 | 3 | 4 | 2 | 9 | 2 | 3 | 1 | 0 | 1 | 3 |
| 2 | 3 | 3 | 3 | 9 | 2 | 2 | 0 | 0 | 1 | 2 |
| 3 | 3 | 2 | 2 | 7 | 2 | 2 | 2 | 1 | 1 | 2 |
| 4 | 3 | 3 | 3 | 9 | 3 | 3 | 1 | 0 | 2 | 3 |
| 5 | 1 | 1 | 1 | 3 | 3 | 1 | 0 | 0 | 0 | 0 |
| 6 | 2 | 3 | 2 | 7 | 3 | 2 | 1 | 1 | 3 | 3 |
| 7 | 3 | 3 | 2 | 8 | 3 | 3 | 1 | 0 | 3 | 3 |
| 8 | 2 | 3 | 1 | 6 | 3 | 3 | 2 | 1 | 2 | 2 |
| 9 | 3 | 0 | 3 | 6 | 0 | 0 | 0 | 0 | 3 | 0 |
| 10 | 3 | 3 | 3 | 9 | 3 | 3 | 1 | 0 | 2 | 3 |
| 11 | 2 | 3 | 1 | 6 | 1 | 3 | 1 | 0 | 2 | 2 |
| 12 | 3 | 3 | 3 | 9 | 2 | 3 | 1 | 1 | 0 | 2 |
| 13 | 3 | 2 | 3 | 8 | 3 | 1 | 0 | 0 | 0 | 2 |
| 14 | 2 | 3 | 1 | 6 | 3 | 3 | 1 | 0 | 0 | 2 |
| 15 | 3 | 1 | 2 | 6 | 3 | 2 | 1 | 0 | 1 | 2 |
| 16 | 3 | 3 | 3 | 9 | 3 | 2 | 3 | 0 | 1 | 2 |
| 17 | 3 | 4 | 3 | 10 | 1 | 3 | 1 | 0 | 2 | 3 |
| 18 | 2 | 3 | 1 | 6 | 3 | 1 | 0 | 0 | 0 | 3 |
| 19 | 3 | 2 | 2 | 7 | 2 | 2 | 0 | 1 | 0 | 1 |
| 20 | 2 | 2 | 1 | 5 | 3 | 2 | 0 | 0 | 0 | 0 |
| 21 | 3 | 3 | 2 | 8 | 3 | 3 | 0 | 2 | 3 | 3 |
| 22 | 3 | 3 | 2 | 8 | 3 | 3 | 0 | 0 | 0 | 2 |
| 23 | 2 | 3 | 1 | 6 | 3 | 3 | 1 | 1 | 1 | 2 |
| 24 | 3 | 4 | 3 | 10 | 2 | 3 | 1 | 0 | 3 | 2 |
| 25 | 2 | 1 | 1 | 4 | 3 | 2 | 0 | 0 | 2 | 0 |
| 26 | 2 | 1 | 1 | 4 | 3 | 2 | 0 | 0 | 1 | 0 |
| 27 | 2 | 1 | 2 | 5 | 3 | 1 | 1 | 1 | 1 | 0 |
| 28 | 3 | 2 | 2 | 7 | 3 | 2 | 0 | 1 | 1 | 2 |
| 29 | 3 | 2 | 3 | 8 | 3 | 3 | 0 | 0 | 3 | 0 |
| 30 | 3 | 4 | 3 | 10 | 1 | 3 | 1 | 0 | 2 | 2 |
| 31 | 3 | 4 | 3 | 10 | 3 | 3 | 1 | 1 | 3 | 3 |
| 32 | 3 | 4 | 3 | 10 | 2 | 3 | 1 | 0 | 3 | 3 |
| 33 | 3 | 1 | 2 | 6 | 3 | 1 | 0 | 0 | 1 | 0 |
| 34 | 3 | 4 | 2 | 9 | 2 | 3 | 1 | 1 | 2 | 3 |
| 35 | 3 | 2 | 2 | 7 | 3 | 3 | 1 | 1 | 1 | 3 |
| 36 | 3 | 2 | 2 | 7 | 3 | 3 | 2 | 0 | 1 | 2 |
| 37 | 3 | 3 | 1 | 7 | 3 | 3 | 2 | 2 | 2 | 3 |

|  |  |  |  |  |  |  |  |  |  |  |
| --- | --- | --- | --- | --- | --- | --- | --- | --- | --- | --- |
| Pathologist 3 |  |  |  |  |  |  |  |  |  |  |
|  |  |  |  |  |  |  |  |  |  |  |
| 1 | 3 | 3 | 2 | 8 | 2 | 2 | 1 | 0 | 0 | 3 |
| 2 | 3 | 2 | 2 | 7 | 2 | 2 | 1 | 0 | 0 | 2 |
| 3 | 3 | 2 | 2 | 7 | 2 | 2 | 1 | 0 | 0 | 2 |
| 4 | 3 | 2 | 2 | 7 | 2 | 2 | 1 | 0 | 1 | 2 |
| 5 | 1 | 0 | 1 | 2 | 1 | 1 | 1 | 0 | 0 | 0 |
| 6 | 3 | 2 | 3 | 8 | 3 | 2 | 1 | 0 | 2 | 2 |
| 7 | 3 | 3 | 3 | 9 | 2 | 2 | 1 | 0 | 2 | 3 |
| 8 | 2 | 3 | 2 | 7 | 2 | 2 | 1 | 0 | 2 | 2 |
| 9 | 3 | 0 | 3 | 6 | 0 | 0 | 0 | 0 | 3 | x |
| 10 | 3 | 4 | 3 | 10 | 3 | 3 | 2 | 0 | 2 | 3 |
| 11 | 3 | 4 | 3 | 10 | 2 | 3 | 3 | 0 | 2 | 3 |
| 12 | 3 | 3 | 3 | 9 | 2 | 2 | 1 | 0 | 2 | 2 |
| 13 | 3 | 2 | 3 | 8 | 2 | 2 | 1 | 1 | 3 | 1 |
| 14 | 3 | 2 | 3 | 8 | 1 | 2 | 1 | 0 | 1 | 2 |
| 15 | 3 | 1 | 2 | 6 | 1 | 1 | 1 | 0 | 1 | 2 |
| 16 | 3 | 4 | 3 | 10 | 2 | 3 | 1 | 0 | 2 | 3 |
| 17 | 3 | 4 | 3 | 10 | 1 | 3 | 1 | 0 | 2 | 3 |
| 18 | 3 | 4 | 3 | 10 | 3 | 3 | 1 | 0 | 1 | 3 |
| 19 | 2 | 3 | 2 | 7 | 2 | 2 | 1 | 0 | 0 | 2 |
| 20 | 3 | 3 | 3 | 9 | 3 | 2 | 1 | 0 | 0 | 3 |
| 21 | 3 | 2 | 3 | 8 | 2 | 3 | 1 | 0 | 1 | 3 |
| 22 | 1 | 2 | 1 | 4 | 2 | 2 | 1 | 0 | 0 | 2 |
| 23 | 3 | 4 | 3 | 10 | 3 | 3 | 3 | 0 | 1 | 2 |
| 24 | 2 | 2 | 2 | 6 | 3 | 2 | 1 | 0 | 2 | 2 |
| 25 | 2 | 2 | 2 | 6 | 3 | 1 | 1 | 0 | 1 | 1 |
| 26 | 3 | 3 | 2 | 8 | 3 | 1 | 1 | 0 | 1 | 2 |
| 27 | 3 | 3 | 2 | 8 | 3 | 1 | 1 | 0 | 1 | 0 |
| 28 | 3 | 4 | 2 | 9 | 3 | 1 | 1 | 0 | 1 | 1 |
| 29 | 3 | 3 | 2 | 8 | 3 | 1 | 1 | 0 | 1 | 1 |
| 30 | 3 | 4 | 3 | 10 | 2 | 3 | 1 | 0 | 3 | 3 |
| 31 | 3 | 3 | 3 | 9 | 2 | 3 | 1 | 0 | 3 | 3 |
| 32 | 3 | 2 | 3 | 8 | 2 | 3 | 1 | 0 | 3 | 2 |
| 33 | 2 | 2 | 2 | 6 | 2 | 2 | 1 | 0 | 1 | 1 |
| 34 | 2 | 2 | 2 | 6 | 1 | 2 | 1 | 0 | 2 | 2 |
| 35 | 3 | 3 | 3 | 9 | 3 | 3 | 2 | 0 | 2 | 3 |
| 36 | 3 | 4 | 3 | 10 | 3 | 2 | 1 | 0 | 2 | 2 |
| 37 | 3 | 3 | 2 | 8 | 2 | 2 | 1 | 0 | 1 | 2 |

ALVAL = aseptic vasculitis-associated lesion
